# The Impact of a Very Short Abstinence Period on Assisted Reproductive Technique Outcomes: A Systematic Review and Meta-Analysis

**DOI:** 10.3390/antiox12030752

**Published:** 2023-03-20

**Authors:** Federica Barbagallo, Rossella Cannarella, Andrea Crafa, Sandro La Vignera, Rosita A. Condorelli, Claudio Manna, Aldo E. Calogero

**Affiliations:** 1 Department of Clinical and Experimental Medicine, University of Catania, 95123 Catania, Italy; 2 Biofertility IVF and Infertility Center, 00198 Rome, Italy; 3 Department of Biomedicine and Prevention, University of Rome “Tor Vergata”, 00133 Rome, Italy

**Keywords:** ejaculatory abstinence, length of sexual abstinence, consecutive ejaculation, assisted reproductive technologies, couples infertility

## Abstract

Background: Previous studies supported the beneficial effects of a very short abstinence period on sperm quality. This systematic review and meta-analysis aimed to evaluate the effects of a very short abstinence period (within 4 h) on assisted reproductive technique (ART) outcomes. Methods: A literature search was performed using the Pubmed, Scopus, Web of Science, and Cochrane databases. A meta-analysis was performed according to the Preferred Reporting Items for Systematic Review and Meta-Analysis Protocols (PRISMA-P) for randomized controlled trials (RCTs). All eligible studies were selected following the PICOS (Population, Intervention, Comparison/Comparator, Outcomes, Study type) model. The following pregnancy outcomes after ART were considered: fertilization rate (FR), implantation rate (IR), clinical pregnancy rate (CPR), live birth rate (LBR), and miscarriage rate (MR). This study was registered on PROSPERO (CRD42023396429). Results: We evaluated 414 records for eligibility, and 7 studies were ultimately included. Our analysis showed that a very short abstinence period significantly increased the IR, CPR, and LBR after ART. No significant differences were found for the FR and MR. Conclusions: A second ejaculation collected very shortly after the first one could represent a simple strategy to improve the results of ART, especially in couples including patients with abnormal sperm parameters.

## 1. Introduction

In 1978, the first in vitro fertilization (IVF) treatment was performed in the United Kingdom, leading to the birth of Louise Brown [1]. Since that time, assisted reproductive technologies (ARTs) have developed and spread around the world to help infertile couples achieve pregnancy [2]. The use of infertility treatments is expected to increase over the coming years due to ongoing social changes, lifestyle changes, and exposure to pollutants. Today, approximately 15% of couples of reproductive age are estimated to be infertile (approximately 48.5 million couples worldwide) [3]. The male factor is responsible for approximately 30% of cases, the female factor for 50%, and both partners are involved in the remaining 20% of cases [3]. Male infertility has long been neglected, and the failure of the ART cycles has been attributed exclusively to the female component.

In recent decades, the attention of the scientific world toward problems related to the male factor of infertility has widely increased. More than 30 million men worldwide are infertile [4]. Furthermore, a progressive decline in sperm quality has been described [5,6]. Although the reasons are still unclear, unhealthy lifestyles and environmental/professional pollution, such as smoking [7], obesity [8], psychological stress [9], and exposure to heavy metals [10], industrial pollutants [11], and endocrine disruptors (EDCs) [12,13,14] may play a major role in decreasing sperm quality. From prenatal life to adulthood, exposure to EDCs can impair male fertility through several mechanisms, such as hormone receptor binding, the dysregulation of receptor expression and steroidogenesis, and epigenetic alterations [14]. Exposure to EDCs has been linked to decreased sperm quality and increased sperm DNA fragmentation (SDF) [14]. In turn, altered sperm parameters can influence the fertilization rate (FR), the cleavage rate (CR), and embryo quality [15]. Furthermore, the latter may be associated with higher rates of aneuploidy and miscarriage [16]. Therefore, better sperm quality may improve ART outcomes. Among the various factors that can influence sperm quality, the length of sexual abstinence is often overlooked, even though its duration has been amply demonstrated to influence sperm parameters.

The World Health Organization (WHO) recommends a sexual abstinence of 2–7 days for semen analysis [17]. Throughout its various editions, published from 1980 until the most recent edition in 2021, the WHO Manual for the Examination and Processing of Semen has undergone many updates to keep up with advances in the field except for the indications regarding the duration of sexual abstinence, which remained unchanged. However, the European Society of Human Reproduction and Embryology (ESHRE) suggests an abstinence period of only 3–4 days [18]. The basis for these indications is uncertain, and the need to modify the current recommendations on the duration of sexual abstinence has been suggested [19,20].

In recent decades, several studies have focused on the opportunity of using a second ejaculation that is collected after a very short period of abstinence in infertile patients. A recent systematic review and meta-analysis published by our group investigated the effects of a very short abstinence period (within 4 h) on conventional sperm parameters and the sperm DNA fragmentation (SDF) rate. The results showed a significant increase in sperm concentration and total and progressive motility in the second ejaculate compared to the first, especially in patients with altered sperm parameters. On the other hand, the SDF rate was significantly reduced in the second collection compared to the first [21]. These results suggested that a second consecutive ejaculation could help to obtain spermatozoa of better quality, especially in male partners with altered sperm parameters. These findings could have important applications in both natural and assisted reproduction. Based on these results, the objective of this systematic review and meta-analysis is to assess the effects of a very short abstinence period (within 4 h) on ART outcomes.

## 2. Materials and Methods

### 2.1. Sources

The present meta-analysis was performed according to the Preferred Reporting Items for Systematic Review and Meta-Analysis Protocols (PRISMA-P) for RCTs [22,23] and the MOOSE guidelines for Meta-analyses and Systematic Reviews of Observational Studies [24]. The PRISMA checklist is included in Table S1, and the MOOSE checklist is included in Supplementary Table S2.

This study was registered in the Internal Prospective Register of Systematic Reviews (PROSPERO) database and has the registration number CRD42023396429.

The articles were selected through an extensive search of the Pubmed, Scopus, Web of Science, and Cochrane Reviews databases from their establishment to January 2023. The search strategy included the combination of several Medical Subjects Headings (MeSH) terms and keywords. For ART, the asterisk operator was used to include both “assisted reproductive technology” and “assisted reproductive technique” at the same time. Similarly, the asterisk operator was used to include both “ejaculate” and “ejaculation” at the same time. The search strings used for each database are shown in Supplementary Table S3. Additionally, manual searches were conducted through the study reference lists. Only articles in English and those conducted on humans were selected. All abstracts and relevant full texts were evaluated. Two authors independently (F.B. and R.C.) reviewed the abstracts and selected only the articles that were pertinent to the aim of this study. Any disagreement was resolved by a discussion with a third investigator (A.E.C.). The reference lists of identified articles were also used to find pertinent studies.

### 2.2. Study Selection

All eligible studies were selected following the PICOS (Population, Intervention, Comparison/Comparator, Outcomes, Study type) model (Table 1) [25]. We considered for inclusion all studies that evaluated the effects of a very short abstinence period (within 4 h) on pregnancy outcome (FR, implantation rate (IR), clinical pregnancy rate (CPR), live birth rate (LBR), and miscarriage rate (MR)) after ART. Case reports, comments, letters to the editor, systematic or narrative reviews, and those studies that did not allow for the extraction of the outcomes of interest were excluded from the analysis.

### 2.3. Data Extraction

The following data were collected: the first author’s name, year of publication, study design, the total number of couples, semen characteristics of enrolled male partners (normozoospermic or altered sperm parameters), abstinence period of first ejaculate, abstinence period of second ejaculate (within 4 h), methods used for semen analysis, type of ART (IVF or intracytoplasmic sperm injection (ICSI)), type of embryo transfer (frozen/fresh cycles), and pregnancy outcomes (FR, IR, CPR, LBR, or MR) after ART.

### 2.4. Quality of Evidence Evaluation

The quality of evidence (QoE) assessment of the articles included in this systematic review and meta-analysis was performed using the Cambridge Quality Checklists [26]. We explain this checklist in detail in the following meta-analysis https://doi.org/10.3390/jcm11247303 [21].

### 2.5. Statistical Analysis

Cochran’s Q test and I^2^ statistics were used to evaluate the statistical heterogeneity. Specifically, if the I^2^ result was lower than or equal to 50%, the variation of the studies was considered to be homogenous, and the fixed effect model was adopted. If the I^2^ was higher than 50%, there was significant heterogeneity between the studies, and the random effects model was used. The pooled effect size and the corresponding CI were calculated after the exclusion of one study at a time. A study resulting in a change of inference upon its exclusion was labeled a “sensitive study”. Publication bias was qualitatively analyzed by the asymmetry of the funnel plot, which suggested some missing studies on one side of the graph. For a quantitative analysis of publication bias, we employed Egger’s intercept test, which evaluated the statistical significance of publication bias. In case of the presence of publication bias, unbiased estimates were calculated using the “trim and fill” method. A *p*-value lower than 0.05 was considered statistically significant. The analysis was performed using RevMan software, v. 5.4 (Cochrane Collaboration, Oxford, UK), and Comprehensive Meta-Analysis Software (Version 3) (Biostat Inc., Englewood, NJ, USA).

## 3. Results

**Figure 1:**
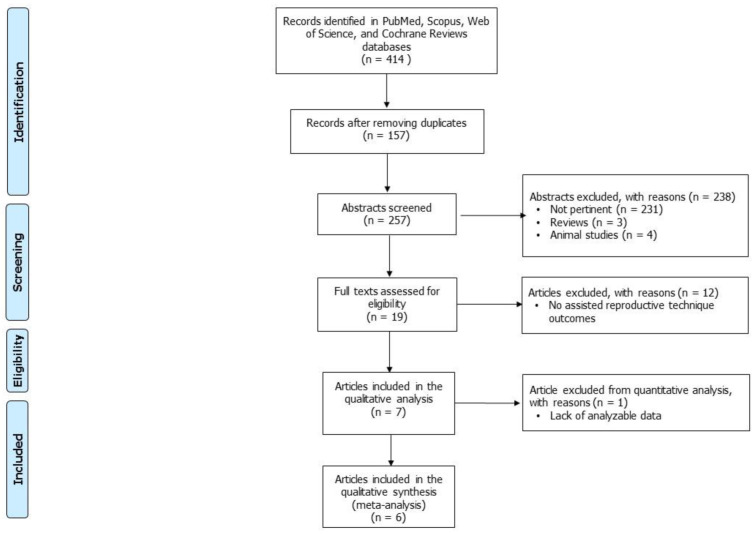
Flowchart of included studies.

The aforementioned search strategy identified 414 records. After the exclusion of 157 duplicates, the remaining 257 articles were screened. Of these articles, 231 were judged not pertinent after reading their titles and abstracts. Three studies were excluded because they were reviews, and two were excluded because they were animal studies. Twelve studies were excluded because they evaluated only the effect of a short period of abstinence on sperm parameters and not on ART outcomes. Finally, seven articles were included in the analysis [27,28,29,30,31,32,33] (Figure 1). All studies were judged to be of moderate quality after assessment with the Cambridge Quality Checklists (Table 2). Table 3 summarizes the main characteristics of the seven studies included in the systematic review and meta-analysis.

### 3.1. Effects of a Very Short Period of Abstinence on ART Outcomes: Qualitative Analysis

Seven studies evaluated the effects of a very short abstinence period on ART outcomes [27,28,29,30,31,32,33]. Among these, three studies included IVF and embryo transfer (ET) cycles [27,28,29], whereas the remaining four studies evaluated ICSI cycles.

Barash et al. evaluated the influence of a consecutive ejaculate collected 2 h after the first one from thirty-nine infertile patients with oligoasthenozoospermia (OA) scheduled for IVF-ET [27]. The authors found a statistically significant increase in the FR (fertilization was defined as the presence of two or more pronuclei), CR (0.6 ± 1 vs. 1.9 ± 1.7, *p* < 0.05), and pregnancy rates (defined as the appearance of an intrauterine gestational sac) for ET when oocytes were incubated with spermatozoa from the second ejaculate compared to the oocytes incubated with spermatozoa from the first ejaculate [27].

Sugiyam et al. also demonstrated an improvement in the FR (fertilization was defined as the presence of two pronuclei with the extraction of the second polar body) with a second ejaculation collected 30–60 min after the first. In detail, their study included 32 IVF-ET cycles performed in 22 couples in which the male partner had previously exhibited OA. Oocytes were equally and randomly assigned for exposure to spermatozoa from the first or second ejaculate [28]. However, embryo scores (5.9 ± 1.4 vs. 6.1 ± 6.1, *p* > 0.05) and CR (90.1% vs. 92.3, *p* > 0.05) were similar between the first and second ejaculates [28].

Shen et al. reported that the IR, CPR, and LBR from IVF significantly increased with ejaculates from a very short abstinence period (1–3 h) compared to 3–7 days of abstinence [29]. They also reported for the first time that the IR, CPR, and LBR were significantly ameliorated using a semen sample after 1–3 h of abstinence in a frozen–thawed cycle rather than a fresh IVF cycle.

Scarselli et al. conducted a prospective study involving 22 couples awaiting ICSI [30]. All male partners with oligoasthenoteratozoospermia (OAT) were asked to collect a second ejaculate 1 h after the first. The oocytes from each female partner were divided into two groups: 121 oocytes were injected with spermatozoa from the first semen collection (Group 1) and 144 oocytes with spermatozoa from the second semen collection (Group 2). Results in terms of the FR, IR, and CPR were similar between the two groups. However, the rate of euploid blastocysts was statistically higher in Group 2 (27.5% vs. 43.6%, *p* = 0.043).

Ciotti et al. performed a retrospective study on 127 couples treated with ICSI [31]. On the basis of the sperm parameters of the male partners, the couples were distinguished in two groups. In detail, the male partners of the Group 1 (75 cycles) had severe OAT, while the male partners of the control group (52 cycles) had normozoospermia or mild OAT. All male partners of Group 1 were asked to collect a second ejaculate 1 h after the first ejaculate. In Group 1, the utilization rate of the first semen collection was 8.6% for the first sample, 25.7% for the first and second samples combined, and 65.7% for the second sample. The authors found no statistically significant difference in the FR, IR, PR/transfer, and embryo quality between the two groups. The MR was significantly higher in Group 1.

We previously described that a second sample, collected 1 h after the first in severe OA patients, allowed for the achievement of a higher CPR and a better embryo quality than in normozoospermic men or mild OA patients. Furthermore, the use of the second ejaculate, collected after 1 h after the first in patients with severe OA, resulted in similar results in terms of the FR, IR, LBR, and MR of couples with normozoospermic or mildly OA male partners [32].

Patel et al. retrospectively analyzed two groups of couples who underwent cycles of ICSI [33]. Both Groups A and B included male partners with severe OAT. For the Group A (n = 56), the first semen sample was used for ICSI cycles, while for Group B (n = 4), a second semen sample collected within a shorter abstinence period of 1 h was used. The FR was similar among the two groups. The grade 1 embryos at day 3 in Group A were 62.8% and 54.3% in group B (*p* = 0.031). However, the authors found an increased percentage of embryos that could be cryopreserved for further use in Group B compared to Group A (37.1% vs. 31.6%, respectively). Similarly, on day 5/day 6, the percentage of embryos that were cryopreserved in Group B was higher than in Group A (50% vs. 32.8%, respectively) [33].

### 3.2. Effects of a Short Period of Abstinence on ART Outcome: Quantitative Analysis

#### 3.2.1. Fertilization Rate

**Figure 2:**
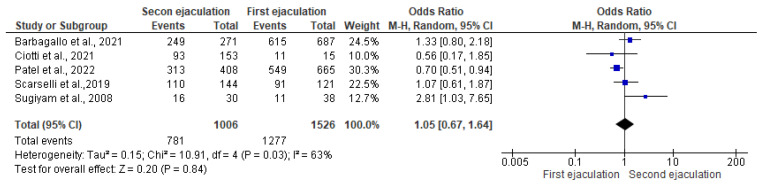
Forest plot showing the effects of a very short abstinence period on fertilization rate [28,30,31,32,33].

The data on the effects of a very short abstinence period on the FR could be extracted from five studies [28,30,31,32,33]. Barash et al. also evaluated the FR. However, they reported the FR only as the mean ± standard deviation, whereas other studies reported the FR as the ratio between the number of fertilized oocytes divided by the number of inseminated oocytes [27]. Therefore, the study conducted by Barash et al. was excluded from the quantitative analysis for evaluating the FR. The four studies included in the quantitative analysis were all conducted in patients with altered sperm parameters. The statistical analysis showed no significant changes in the FR after a very short abstinence period (OR 1.05 (0.67, 1.64); *p* = 0.84) (Figure 2). This analysis showed the presence of inter-study heterogeneity (Chi^2^ *p* = 0.03, I^2^ = 63%) and therefore, the random model was used. There was no evidence of publication bias, as shown by Egger’s test (*p*-value = 0.11) and funnel plot symmetry (Supplementary Figure S1A). Neither study was sensitive enough to change the above results (Supplementary Figure S1B).

#### 3.2.2. Implantation Rate

**Figure 3:**
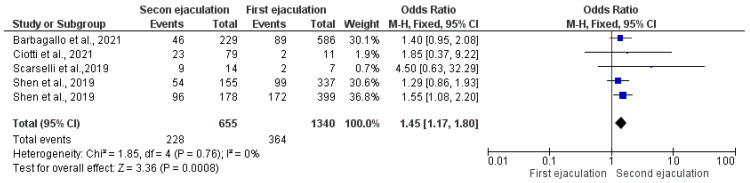
Forest plot of studies showing the effects of a very short abstinence period on implantation rate [29,30,31,32].

Four studies investigated the effects of a very short abstinence period on the IR [29,30,31,32]. Ciotti et al. [31] and Barbagallo et al. [32] calculated the IR as the number of sacs identified on ultrasound divided by the number of transferred embryos. Shen et al. [29] and Scarselli et al. [30] did not specify how the IR was calculated. The study by Shen and colleagues was considered twice because the authors distinguished between fresh and frozen–thawed cycles [29]. The statistical analysis showed a statistically significant improvement in the IR after a very short abstinence period (OR 1.45 (1.17, 1.80); *p* = 0.0008) (Figure 3). Inter-study heterogeneity was not observed in this analysis (Chi^2^ = 1.85, I^2^ = 0%). There was no evidence of publication bias, as shown by Egger’s test (*p*-value = 0.08) and the symmetry of the funnel plots (Supplementary Figure S2A). No studies were sensitive enough to alter the above results (Supplementary Figure S2B).

#### 3.2.3. Clinical Pregnancy Rate

**Figure 4:**
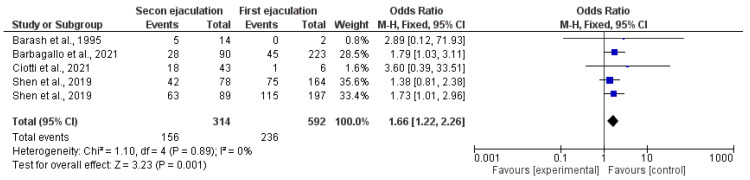
Forest plot showing the effects of a very short abstinence period on the clinical pregnancy rate [27,29,31,32].

Five studies evaluated the effects of a very short abstinence period on the CPR [27,29,30,31,32]. In particular, Barash et al. [27] defined the PR as the rate between the number of ultrasonographic views of one or more gestational sacs divided by the number of ETs. Scarselli et al. [30] did not specify the definition of PR used. Shen et al. [29] reported that the CPR was defined as ultrasound evidence of intrauterine fetal heartbeat at 7 weeks, but they did not specify how they calculated the rate. Ciotti et al. [31] reported the CPR as the number of clinical pregnancies (evidence of a gestational sac on ultrasound) divided by the number of ET cycles. Finally, Barbagallo et al. [32] defined CPR as the number of pregnancies with a fetal heartbeat divided by the number of pick-ups with at least one oocyte retrieved. Again, the study by Shen and colleagues was considered twice because the authors distinguished between fresh and frozen–thawed cycles [29]. The analysis showed a statistically significant improvement in the CPR after a very short abstinence period (OR 1.70 (1.26, 2.21); *p* = 0.0006) (Figure 4). Inter-study heterogeneity was not observed in this analysis (Chi^2^ = 2.07, I^2^ = 0%). There was evidence of publication bias, as shown by Egger’s test (*p*-value = 0.03) and the asymmetry of the funnel plot (Supplementary Figure S3A). No studies were sensitive enough to alter the above results (Supplementary Figure S3B).

#### 3.2.4. Live Birth Rate

**Figure 5:**
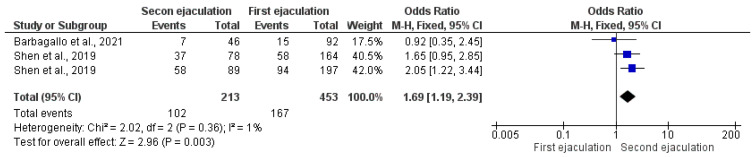
Forest plot showing the effects of a very short abstinence period on the live birth rate [29,32].

Only two studies evaluated the effects of a very short abstinence period on the LBR [29,32]. Barbagallo et al. [32] evaluated the LBR as the number of deliveries with at least one live birth divided by the number of pick-ups with at least one oocyte retrieved, while the study of Shen et al. [29], did not report how the LBR was calculated. Regarding other outcomes, the study by Shen and colleagues was considered twice because they distinguished between fresh and frozen–thawed cycles [29]. The statistical analysis showed a statistically significant improvement in the LBR after a very short abstinence period (OR 1.69 (1.19, 2.39); *p* = 0.003) (Figure 5). Inter-study heterogeneity was not observed in this analysis (Chi^2^ = 2.02, I^2^ = 1%). There was no evidence of publication bias, as shown by Egger’s test (*p*-value = 0.10) and the funnel plot symmetry (Supplementary Figure S4A). One study [29] was sensitive enough to alter the above results (OR 1.44 (0.89, 2.31); *p* = 0.139) (Supplementary Figure S4B).

#### 3.2.5. Miscarriage Rate

**Figure 6:**
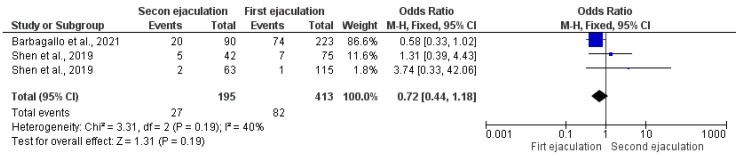
Forest plot of studies showing the effects of a very short abstinence period on the miscarriage rate [29,32].

Only two studies evaluated the effects of a very short abstinence period on the MR [29,32]. In the study conducted by Barbagallo et al. [32], the MR was calculated as the number of spontaneous abortions divided by the total number of pregnancies. Shen et al. [29] did not report how the LBR was calculated. The study by Shen and colleagues was considered twice because the authors distinguished between fresh and frozen–thawed cycles [29]. The statistical analysis showed no statistically significant change in the MR after a very short abstinence period (OR 0.72 (0.44, 1.18); *p* = 0.19) (Figure 6). Inter-study heterogeneity was not observed in this analysis (Chi^2^ =3.31, I^2^ = 40%). There was evidence of publication bias, as shown by Egger’s test (*p*-value = 0.04) and the funnel plot asymmetry (Supplementary Figure S5A). However, no study was sensitive enough to alter the above results (Supplementary Figure S5B).

## 4. Discussion

**Figure 7:**
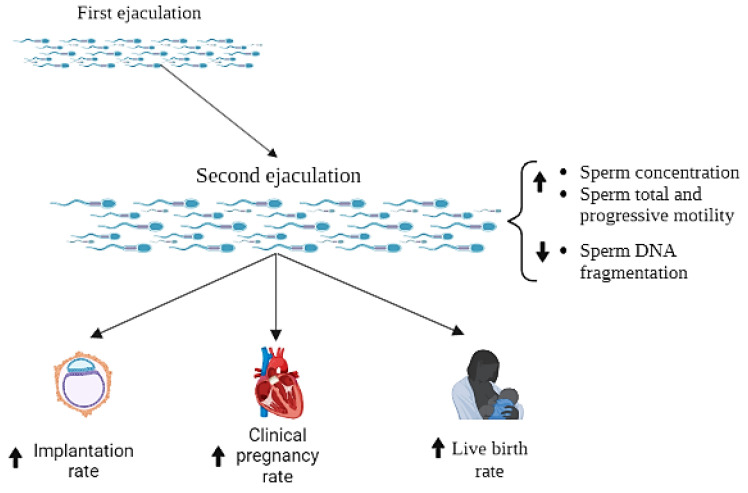
Effects of a second ejaculation performed after a very short period of abstinence from the first on sperm quality and assisted reproductive technique outcomes.

To our knowledge, this systematic review and meta-analysis is the first to assess the influence of a very short abstinence period on ART outcomes. The quantitative analysis supported our hypothesis and showed that a very short abstinence period significantly increased the IR, CPR, and LBR in couples undergoing ART, while no significant changes were found for the FR and MR (Figure 7).

In recent decades, due to the growing number of infertile couples undergoing ART, a significant number of studies have focused on the effects of the length of sexual abstinence on the outcome of these techniques. A recent meta-analysis conducted by Li and colleagues compared the effects of short (less than 4 days) and long (4–7 days) sexual abstinence periods on the clinical outcomes of fresh ET cycles after ART [34]. This meta-analysis indicated that a shorter abstinence period could lead to a higher IR (overall OR = 1.39; 95% CI (1.17, −1.65); *p* = 0.0001) and pregnancy rate (overall OR = 1.44; 95% CI (1.17, −1.78); *p* = 0.0006) for patients undergoing ART [34]. In parallel, an increasing number of researchers have argued for the potential improvement of sperm quality in a second ejaculate collected after a very short abstinence period (within few hours) from the first collection. In a recent systematic review and meta-analysis, we demonstrated the positive effects of a very short abstinence period on sperm quality, especially in patients with abnormal sperm parameters [21]. To date, few studies have reviewed the effects of a very short abstinence period on not only sperm quality but also the clinical outcomes of ART.

**Figure 8:**
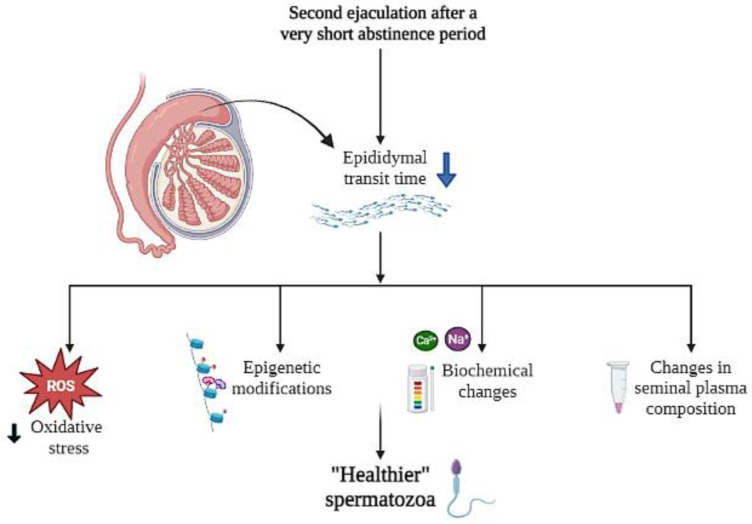
Mechanism(s) of improved sperm quality in the second ejaculate collected after a very short period of abstinence (within 4 h).

The mechanism(s) of the improvement in sperm quality in the second ejaculate collected after a short interval is unclear. Several hypotheses have been put forward to explain the observed improvement in sperm parameters. These include epigenetic modifications [29,35], biochemical changes between consecutive ejaculations [36], and seminal plasma modifications [37] (Figure 8).

A short period of abstinence could reduce the time of spermatozoa transit through the epididymis and, in turn, result in a shorter length of exposure for the spermatozoa to reactive oxygen species (ROS) in the cauda epididymis. This can reduce the damaging effects of oxidative stress on spermatozoa and can lead to a “younger” and “healthier” sperm population. Furthermore, epigenetic modifications occur during epididymal transit. Different epigenetic modifications have been described in ejaculates collected after 1–3 h of abstinence compared to those collected after 3–7 days [29]. In detail, sperm butyryl-lysine, propionyl-lysine, and malonyl-lysine modifications were found significantly decreased and trimethyl-lysine modifications significantly increased after a shorter abstinence length [29]. It was also hypothesized that the decrease in Na^+^ and Ca^2+^ concentrations and of the cytoplasmic pH in spermatozoa from the caput to the cauda of the epididymis may have a role in the improvement of sperm motility in the second ejaculate [36]. Furthermore, a recent study compared the metabolomic profile of seminal plasma in two consecutive ejaculates, showing an increase in the absolute amount of pyruvate (one of the most important energy sources for sperm mitochondrial function and motility) and taurine (which has antioxidant and membrane stabilizing effects) in spermatozoa from the second ejaculate [37].

A short period of abstinence could lead to the ejaculation of “younger” and “healthier” spermatozoa that have a shorter length of exposure to the toxic effects of ROS in the cauda epididymis. Ample evidence has now been provided that oxidative stress (OS) plays an important role in the pathogenesis of male infertility. Seminal ROS, mainly produced by leukocytes or abnormal spermatozoa, are natural products of metabolic pathways and are also essential for some physiological functions, such as sperm hyperactivation and capacitation, but only in small physiological quantities. Excessive ROS production causes an imbalance between the oxidative and antioxidant systems, resulting in an increased OS, which can negatively affect male fertility through several mechanisms [38]. Human spermatozoa are very sensitive to the damage caused by ROS due to their high content of polyunsaturated fatty acids (PUFAs). Furthermore, human spermatozoa are also characterized by a limited number of endogenous repair mechanisms. Therefore, OS can damage the sperm membrane and DNA and, in turn, impair the ability of spermatozoa to fertilize oocytes [38]. Thus, a different epididymal transit time might play a major role in the better sperm quality observed in a second consecutive ejaculate compared to the first. Intriguingly, the time of spermatozoa transit through the epididymis is not always the same. It was described that the epididymal spermatozoa transit time was three times longer in oligozoospermic patients than in normozoospermic men [39]. Thus, spermatozoa from patients with severe OA remain in the genital tract for a prolonged time and, in turn, are more exposed to the harmful effects of OS. This could explain the higher improvement in sperm quality after a very short period of abstinence in patients with altered sperm parameters compared with normozoospermic men. According to this hypothesis, the quantitative proteomic analysis showed that the proteins overexpressed in spermatozoa of the second ejaculate are implicated in physiological functions, such as sperm motility, capacitation, and antioxidant defense [29]. Furthermore, Shen and colleagues reported a greater total antioxidant capacity in ejaculates obtained from short (1–3 h) versus longer (3–7 days) abstinence periods [29]. Mayorga-Torres and colleagues described a reduction of ROS levels in four repeated ejaculations at two-hour intervals, with a significant difference in the fourth collection compared to the first one [40].

This hypothesis is further supported by the decrease in the SDF rate in semen samples collected after a very short abstinence period [21]. The SDF rate may be associated with defective maturation, apoptosis, and OS [41]. A high SDF in couples undergoing ART is associated with worse outcomes and a lower LBR [42]. A higher SDF also correlates with worse outcomes in ICSI cycles of couples with non-male-factor infertility, suggesting that defects in sperm may be hidden and can significantly affect ART outcomes [43]. The last edition of the WHO Manual for Semen Analysis recognized the importance of the SDF test, which could provide important additional information on sperm quality in the male infertility workup [17]. Thus, adequate an diagnostic and therapeutic workup of male partners aimed at reducing the rate of SDF before ART is essential to improve success rates. Sperm DNA integrity is a prerequisite for successful fertilization, embryo development, implantation, and pregnancy [44]. Consequently, previous studies have shown an improvement in both SDF and embryo quality after a very short abstinence period [21]. Similarly, Scarselli et al. found a higher rate of euploid blastocysts with ejaculates collected after a one-hour abstinence length compared to ejaculates collected after a conventional abstinence period [30]. The authors speculated that the significant increase in euploid blastocysts obtained in the group with a very short abstinence period could be explained with normal protamination in one hour of abstinence. In fact, in the same study, the authors also showed a higher percentage of mature chromatin in Group 2 spermatozoa [30]. Among the studies evaluating ART outcomes, Shen and colleagues reported for the first time that the IR, CPR, and LBR were significantly increased using a semen sample obtained after 1–3 h of abstinence in a frozen–thawed cycle rather than fresh IVF cycles [29]. Therefore, Shen and colleagues speculated that frozen–thawed cycles may show improved sperm-related embryo quality and reproductive outcomes after IVF [29]. Previous studies have already suggested that elective frozen embryo transfer (eFET) could improve the reproductive outcomes of ART because of the impaired endometrial receptivity in the latter after controlled ovarian stimulation (COS) [45]. In fact, COS with exogenous gonadotropins is linked to impaired endometrial development that may, in turn, alter endometrial receptivity in cycles of ART through different mechanisms resulting in embryo–endometrium asynchrony [45]. However, to date, low quality evidence indicated that LBRs are increased with the use of frozen compared to fresh ET in all patients undergoing ART, and the use of indiscriminate eFET is not indicated but should be individualized for each patient [46].

As previously shown, better sperm quality improves the fertilization rate and embryo quality [28]. This can be of great value in infertile couples with older female partners. In these cases, the selection of spermatozoa with a lower rate of DNA damage may be helpful, considering the reduced DNA repair capacity of the oocytes of older women [47]. Furthermore, a direct relationship between SDF and aneuploidy rates has also been found in normozoospermic men [48].

The studies included in this meta-analysis were considered to be of moderate quality in the quality of evidence assessment. Nonetheless, some limitations should be taken into consideration. First of all, no RCTs have been conducted on the topic. We must also consider that it is not easy to design an RCT on this topic because it is difficult to define the control group. A more correct experimental model could be to assess the outcomes of ART using spermatozoa from the first and second ejaculate by splitting the oocytes when they are retrieved in an adequate number. The comparison should be made between fertilized oocytes with the first and second ejaculate of the same woman. In this way, we could eliminate important biases provided by the female factor that have a relevant effect on ART outcomes. At the same time, we could reduce most of the biases from the male component. Moreover, a large heterogeneity was found among the included studies. This heterogeneity between studies could be explained by the retrospective design of many studies, a lack of standardization regarding the ART procedure used, and the definition of ART outcomes. Moreover, more data were extracted from a single study. In detail, the study conducted by Shen and colleagues [29] was considered twice in the quantitative analysis for the IR, CPR, LBR, and MR because the authors distinguished between fresh and frozen–thawed cycles. Finally, many of the included studies have a relatively small sample size.

## 5. Conclusions

This is the first systematic review and meta-analysis to evaluate the effects of a very short abstinence period on ART outcomes. Notably, our analysis showed a significantly positive impact of a very short abstinence period on the IR, CPR, and LBR. With the widespread use of ART, improving sperm quality has become essential to achieving better ART success rates. Therefore, optimizing the duration of the abstinence length, in combination with a proper diagnostic and therapeutic workup of infertile couples before they undergo ART, can help increase in the success rate.

## 6. Future Directions

For IVF-ICSI cycles, these findings suggest that the collection of a consecutive ejaculate after a very short period of time from the first could represent a simple strategy to maximize the quality of spermatozoa to be injected for oocyte fertilization and thus improve ART outcomes, particularly in patients with abnormal sperm parameters.

## Figures and Tables

**Table 1:** PICOS (Population, Intervention, Comparison/Comparator, Outcomes, Study type) inclusion criteria.

	Inclusion	Exclusion
Population	Human male in reproductive age	/
Intervention	Short second ejaculation (within 4 h)	Second ejaculation > 4 h
Comparison	Ejaculation after a period of sexual abstinence between 2–7 days	/
Outcome	ART outcome: FR, IR, CPR, LBR, MR	/
Study type	Observational, cohort, cross-sectional, case-control, RCTs	Case reports; comments; letters to the editor; systematic or narrative reviews; in vitro studies; studies on animals

Abbreviations. ART—assisted reproductive techniques; FR—fertilization rate; IR—implantation rate; CPR—clinical pregnancy rate; LBR—live birth rate; MR—miscarriage rate; RCTs—randomized controlled trials.

**Table 2:** Evaluation of study quality using “The Cambridge Quality Checklists”.

Authors and Year of Publication	Checklist for Correlates	Checklist for Risk Factors	Checklist for Casual Risk Factors	Total
Barash et al., 1995 [27]	1	3	6	10/15
Sugiyam et al., 2008 [28]	1	3	6	10/15
Shen et al., 2019 [29]	2	3	6	11/15
Scarselli et al., 2019 [30]	2	3	6	11/15
Ciotti et al., 2021 [31]	1	2	6	9/15
Barbagallo et al., 2021 [32]	2	2	6	10/15
Patel et al., 2022 [33]	1	2	6	9/15

**Table 3:** Effects of a very short abstinence period (within 4 h) on assisted reproductive technique outcomes.

Authors & Years	Study Design	Couples Enrolled	Semen Characterstics of Male Partners	Type of ART	Abstinence Period of First Ejaculate	Abstinence Period of Second Ejaculate	Method Used for Semen Analysis	ET	ART Outcome Assessed	Main Findings
Barash et al., 1995 [27]	Prospective study	n = 39	OA	IVF	3 days	2h	NA	Fresh	FR *No.* of Embryo CPR per ET	↑ FR ↑ no. of Embryo ↑ CPR
Sugiyam et al., 2008 [28]	Prospective study	n = 22	OA	IUI IVF	3–5 days	30–60 min	NA	Fresh	FR Embryo quality CPR per ET	↑ FR - Embryo quality - CPR per ET
Shen et al., 2019 [29]	Prospective study	n = 167 (experimental couples)	N	IVF	3–7 days	1–3 h	WHO, 2010	Fresh	IR CPR Early miscarriage LBR	- IR - CPR - Early miscarriage - LBR
		n = 361 (control couples)	N	IVF	3–7 days	/		Frozen	IR CPR Early miscarriage LBR	↑ IR ↑ CPR - Early miscarriage ↑ LBR
Scarselli et al., 2019 [30]	Prospective study	n = 22	OAT	ICSI	2–5 days	1 h	WHO, 2010	Frozen	FR Euploid blastocyst CPR IR	- FR ↑ Euploid blastocyst - CPR - IR
Ciotti et al., 2021 [31]	Retrospective study	n = 116	Study group 1 = severe OAT (n = 75 cycles)	ICSI	2–3 days	2 h	NA	Fresh	FR IR CPR LBR MR Embryo quality	- FR - IR - CPR - LBR - MR - Embryo quality
			Control Group 0 = N or mild OAT (n = 52 cycles)		2–3 days	/				
Barbagallo et al., 2021 [32]	Retrospective study	n = 313	Group 1 = N or mild OA (n = 223)	ICSI	2–7 days	/	WHO, 2010	Fresh	FR IR CPR LBR MR Embryo quality	- FR - IR ↑ CPR - LBR - MR ↑ Embryo quality
			Group 2 = severe OA (n = 90)		2–7 days	1 h				
Patel et al., 2022 [33]	Retrospective study	n = 97	Group A = OAT (n = 56)	ICSI	2–7 days	/	WHO, 2010	/	FR Embryo 1 grade	- FR ↓ Embryo 1 grade
			Group B = OAT (n = 41)		2–7 days	1 h				

**Abbreviations:** ↓ = reduction; ↑ = increase; - = no significant changes; NA = not available; N = normozoospermic; OA = oligoasthenozoospermic; OAT = oligo-astheno-teratozoospermia; WHO = World Health Organization; IUI = intrauterine insemination; ICSI = intracytoplasmic sperm injection; IVF = in vitro fertilization; ET = embryo transfer; FR = fertilization rate; IR = implantation rate; CPR = clinical pregnancy rate; LBR = live birth rate; MR = miscarriage rate.

## Supplementary Materials

The following supporting information can be downloaded at: https://www.mdpi.com/article/10.3390/antiox12030752/s1: Table S1: PRISMA checklist [22]; Table S2: MOOSE checklist [24]; Table S3: Specific search strings used for each database queried; Figure S1: Funnel plot (A) and sensitivity analysis (B) of studies that evaluated the effects of a very short abstinence period on fertilization rate; Figure S2: Funnel plot (A) and sensitivity analysis (B) of studies that evaluated the effects of a very short abstinence period on implantation rate; Figure S3: Funnel plot (A) and sensitivity analysis (B) of studies that evaluated the effects of a very short abstinence period on clinical pregnancy rate; Figure S4: Funnel plot (A) and sensitivity analysis (B) of studies that evaluated the effects of a very short abstinence period on clinical pregnancy rate; Figure S5: Funnel plot (A) and sensitivity analysis (B) of studies that evaluated the effects of a very short abstinence period on miscarriage rate.

## References

[1] Steptoe P.C., Edwards R.G. (1978). Birth after the reimplantation of a human embryo. Lancet.

[2] Wang J., Sauer M.V. (2006). In vitro fertilization (IVF): A review of 3 decades of clinical innovation and technological advancement. Ther. Clin. Risk Manag..

[3] Crafa A., Calogero A.E., Cannarella R., Mongioi’ L.M., Condorelli R.A., Greco E.A., Aversa A., La Vignera S. (2021). The Burden of Hormonal Disorders: A Worldwide Overview With a Particular Look in Italy. Front. Endocrinol..

[4] Agarwal A., Mulgund A., Hamada A., Chyatte M.R. (2015). A unique view on male infertility around the globe. Reprod. Biol. Endocrinol..

[5] Davidson L.M., Millar K., Jones C., Fatum M., Coward K. (2015). Deleterious effects of obesity upon the hormonal and molecular mechanisms controlling spermatogenesis and male fertility. Hum. Fertil..

[6] Cannarella R., Condorelli R.A., Gusmano C., Barone N., Burrello N., Aversa A., Calogero A.E., La Vignera S. (2021). Temporal Trend of Conventional Sperm Parameters in a Sicilian Population in the Decade 2011–2020. J. Clin. Med..

[7] Condorelli R.A., La Vignera S., Giacone F., Iacoviello L., Vicari E., Mongioi’ L., Calogero A.E. (2013). In vitro effects of nicotine on sperm motility and bio-functional flow cytometry sperm parameters. Int. J. Immunopathol. Pharmacol..

[8] Barbagallo F., Condorelli R.A., Mongioì L.M., Cannarella R., Cimino L., Magagnini M.C., Crafa A., La Vignera S., Calogero A.E. (2021). Molecular Mechanisms Underlying the Relationship between Obesity and Male Infertility. Metabolites.

[9] Leisegang K., Dutta S. (2021). Do lifestyle practices impede male fertility?. Andrologia.

[10] Calogero A.E., Fiore M., Giacone F., Altomare M., Asero P., Ledda C., Romeo G., Mongioì L.M., Copat C., Giuffrida M. (2021). Exposure to multiple metals/metalloids and human semen quality: A cross-sectional study. Ecotoxicol. Environ. Saf..

[11] Perrone P., Lettieri G., Marinaro C., Longo V., Capone S., Forleo A., Pappalardo S., Montano L., Piscopo M. (2022). Molecular Alterations and Severe Abnormalities in Spermatozoa of Young Men Living in the “Valley of Sacco River” (Latium, Italy): A Preliminary Study. Int. J. Environ. Res. Public Health.

[12] Barbagallo F., Condorelli R.A., Mongioì L.M., Cannarella R., Aversa A., Calogero A.E., La Vignera S. (2020). Effects of Bisphenols on Testicular Steroidogenesis. Front. Endocrinol..

[13] Ješeta M., Navrátilová J., Franzová K., Fialková S., Kempisty B., Ventruba P., Žáková J., Crha I. (2021). Overview of the Mechanisms of Action of Selected Bisphenols and Perfluoroalkyl Chemicals on the Male Reproductive Axes. Front. Genet..

[14] Cannarella R., Gül M., Rambhatla A., Agarwal A. (2023). Temporal decline of sperm concentration: Role of endocrine disruptors. Endocrine.

[15] Loutradi K.E., Tarlatzis B.C., Goulis D.G., Zepiridis L., Pagou T., Chatziioannou E., Grimbizis G.F., Papadimas I., Bontis I. (2006). The effects of sperm quality on embryo development after intracytoplasmic sperm injection. J. Assist. Reprod. Genet..

[16] Puscheck E.E., Jeyendran R.S. (2007). The impact of male factor on recurrent pregnancy loss. Curr. Opin. Obstet. Gynecol..

[17] World Health Organization (2021). WHO Laboratory Manual for the Examination and Processing of Human Semen.

[18] Kvist U., Björndahl L. (2002). Manual on Basic Semen Analysis.

[19] Levitas E., Lunenfeld E., Weiss N., Friger M., Har-Vardi I., Koifman A., Potashnik G. (2005). Relationship between the duration of sexual abstinence and semen quality: Analysis of 9,489 semen samples. Fertil. Steril..

[20] Henkel R. (2012). Sperm preparation: State-of-the-art–physiological aspects and application of advanced sperm preparation methods. Asian J. Androl..

[21] Barbagallo F., Cannarella R., Crafa A., Manna C., La Vignera S., Condorelli R.A., Calogero A.E. (2022). The Impact of a Very Short Abstinence Period on Conventional Sperm Parameters and Sperm DNA Fragmentation: A Systematic Review and Meta-Analysis. J. Clin. Med..

[22] Page M.J., McKenzie J.E., Bossuyt P.M., Boutron I., Hoffmann T.C., Mulrow C.D., Shamseer L., Tetzlaff J.M., Akl E.A., Brennan S.E. (2021). The PRISMA 2020 statement: An updated guideline for reporting systematic reviews. BMJ.

[23] Shamseer L., Moher D., Clarke M., Ghersi D., Liberati A., Petticrew M., Shekelle P., Stewart L.A., PRISMA-P Group (2015). Preferred reporting items for systematic review and meta-analysis protocols (PRISMA-P) 2015: Elaboration and explanation. BMJ.

[24] Stroup D.F., Berlin J.A., Morton S.C., Olkin I., Williamson G.D., Rennie D., Moher D., Becker B.J., Sipe T.A., Thacker S.B. (2000). Meta-Analysis of Observational Studies in Epidemiology. A Proposal for Reporting. JAMA.

[25] da Costa Santos C.M., de Mattos Pimenta C.A., Nobre M.R. (2007). The PICO strategy for the research question construction and evidence search. Rev. Lat.-Am. Enferm..

[26] Murray J., Farrington D.P., Eisner M.P. (2009). Drawing conclusions about causes from systematic reviews of risk factors: The Cambridge Quality Checklists. J. Exp. Criminol..

[27] Barash A., Lurie S., Weissman A., Insler V. (1995). Comparison of sperm parameters, in vitro fertilization results, and subsequent pregnancy rates using sequential ejaculates, collected two hours apart, from oligoasthenozoospermic men. Fertil. Steril..

[28] Sugiyam R., Al-Salem J.A., Nishi Y., Sugiyama R., Shirai A., Inoue M., Irahara M. (2008). Improvement of sperm motility by short interval sequential ejaculation in oligoasthenozoospermic patients. Arch. Med. Sci..

[29] Shen Z.Q., Shi B., Wang T.R., Jiao J., Shang X.J., Wu Q.J., Zhou Y.M., Cao T.F., Du Q., Wang X.X. (2019). Characterization of the Sperm Proteome and Reproductive Outcomes with in Vitro, Fertilization after a Reduction in Male Ejaculatory Abstinence Period. Mol. Cell. Proteom..

[30] Scarselli F., Cursio E., Muzzì S., Casciani V., Ruberti A., Gatti S., Greco P., Varricchio M.T., Minasi M.G., Greco E. (2019). How 1 h of abstinence improves sperm quality and increases embryo euploidy rate after PGT-A: A study on 106 sibling biopsied blastocysts. J. Assist. Reprod. Genet..

[31] Ciotti P.M., Calza N., Zuffa S., Notarangelo L., Nardi E., Damiano G., Cipriani L., Porcu E. (2021). Two subsequent seminal productions: A good strategy to treat very severe oligoasthenoteratozoospermic infertile couples. Andrology.

[32] Barbagallo F., Calogero A.E., Condorelli R.A., Farrag A., Jannini E.A., La Vignera S., Manna C. (2021). Does a Very Short Length of Abstinence Improve Assisted Reproductive Technique Outcomes in Infertile Patients with Severe Oligo-Asthenozoospermia?. J. Clin. Med..

[33] Patel D.V., Patel T., Maheshwari N., Soni S., Patel R.G. (2022). Retrospective Analysis of the First Collection versus the Second Collection in Severe Oligo-asthenoteratozoospermia Cases in Self-Intracytoplasmic Sperm Injection Patients. J. Hum. Reprod. Sci..

[34] Li J., Shi Q., Li X., Guo J., Zhang L., Quan Y., Ma M., Yang Y. (2020). The Effect of Male Sexual Abstinence Periods on the Clinical Outcomes of Fresh Embryo Transfer Cycles Following Assisted Reproductive Technology: A Meta-Analysis. Am. J. Mens Health.

[35] Sharma U., Conine C.C., Shea J.M., Boskovic A., Derr A.G., Bing X.Y., Belleannee C., Kucukural A., Serra R.W., Sun F. (2016). Biogenesis and function of tRNA fragments during sperm maturation and fertilization in mammals. Science.

[36] Bahadur G., Almossawi O., Zeirideen Zaid R., Ilahibuccus A., Al-Habib A., Muneer A., Okolo S. (2016). Semen characteristics in consecutive ejaculates with short abstinence in subfertile males. Reprod. Biomed. Online.

[37] Alipour H., Duus R.K., Wimmer R., Dardmeh F., Du Plessis S.S., Jørgensen N., Christiansen O.B., Hnida C., Nielsen H.I., Van Der Horst G. (2021). Seminal plasma metabolomics profiles following long (4–7 days) and short (2 h) sexual abstinence periods. Eur. J. Obstet. Gynecol. Reprod. Biol..

[38] Agarwal A., Parekh N., Panner Selvam M.K., Henkel R., Shah R., Homa S.T., Ramasamy R., Ko E., Tremellen K., Esteves S. (2019). Male Oxidative Stress Infertility (MOSI): Proposed Terminology and Clinical Practice Guidelines for Management of Idiopathic Male Infertility. World J. Mens Health.

[39] Johnson L., Varner D.D. (1988). Effect of daily spermatozoan production but not age on transit time of spermatozoa through the human epididymis. Biol. Reprod..

[40] Mayorga-Torres B.J., Camargo M., Agarwal A., du Plessis S.S., Cadavid Á.P., Cardona Maya W.D. (2015). Influence of ejaculation frequency on seminal parameters. Reprod. Biol. Endocrinol..

[41] Agarwal A., Majzoub A., Baskaran S., Panner Selvam M.K., Cho C.L., Henkel R., Finelli R., Leisegang K., Sengupta P., Barbarosie C. (2020). Sperm DNA Fragmentation: A New Guideline for Clinicians. World J. Mens Health.

[42] Osman A., Alsomait H., Seshadri S., El-Toukhy T., Khalaf Y. (2015). The effect of sperm DNA fragmentation on live birth rate after IVF or ICSI: A systematic review and meta-analysis. Reprod. Biomed. Online.

[43] Borges E., Zanetti B.F., Setti A.S., Braga D.P.A.F., Provenza R.R., Iaconelli A. (2019). Sperm DNA fragmentation is correlated with poor embryo development, lower implantation rate, and higher miscarriage rate in reproductive cycles of non-male factor infertility. Fertil. Steril..

[44] Ward W.S. (2010). Function of sperm chromatin structural elements in fertilization and development. Mol. Hum. Reprod..

[45] Shapiro B.S., Daneshmand S.T., Garner F.C., Aguirre M., Hudson C., Thomas S. (2011). Evidence of impaired endometrial receptivity after ovarian stimulation for in vitro fertilization: A prospective randomized trial comparing fresh and frozen-thawed embryo transfer in normal responders. Fertil. Steril..

[46] Roque M., Haahr T., Geber S., Esteves S.C., Humaidan P. (2019). Fresh versus elective frozen embryo transfer in IVF/ICSI cycles: A systematic review and meta-analysis of reproductive outcomes. Hum. Reprod. Update.

[47] Fernández-Díez C., González-Rojo S., Lombó M., Herráez M.P. (2016). Impact of sperm DNA damage and oocyte-repairing capacity on trout development. Reproduction.

[48] Vendrell X., Ferrer M., García-Mengual E., Muñoz P., Triviño J.C., Calatayud C., Rawe V.Y., Ruiz-Jorro M. (2014). Correlation between aneuploidy, apoptotic markers and DNA fragmentation in spermatozoa from normozoospermic patients. Reprod. Biomed. Online.

